# Interstitial High-Dose-Rate Brachytherapy Combined with External Beam Radiation Therapy for Dose Escalation in the Primary Treatment of Locally Advanced, Non-Resectable Superior Sulcus (Pancoast) Tumors: Results of a Monocentric Retrospective Study

**DOI:** 10.3390/jcm13247550

**Published:** 2024-12-11

**Authors:** Maria Neu, Klaus-Henning Kahl, Melina Körner, Renate Walter, Stephan Raab, Bertram Jehs, Lukas Käsmann, Vratislav Strnad, Georg Stüben, Nikolaos Balagiannis

**Affiliations:** 1 Department of Radiotherapy and Radiation Oncology, Faculty of Medicine, University of Augsburg, 86156 Augsburg, Germany; 2 Comprehensive Cancer Center Augsburg (CCCA), Faculty of Medicine, University of Augsburg, 86156 Augsburg, Germany; 3 Comprehensive Cancer Center Alliance WERA (CCC WERA), 86156 Augsburg, Germany; 4 Bavarian Cancer Research Center (BZKF), 86156 Augsburg, Germany; 5 Department of Radiation Protection and Medical Physics, Faculty of Medicine, University of Augsburg, 86156 Augsburg, Germany; 6 Department of Thoracic Surgery, Faculty of Medicine, University of Augsburg, 86156 Augsburg, Germany; 7 Department of Diagnostic and Interventional Radiology and Neuroradiology, Faculty of Medicine, University of Augsburg, 86156 Augsburg, Germany; 8 Department of Radiation Oncology, University Hospital, LMU Munich, 80539 Munich, Germany; 9 Department of Radiation Oncology, University Hospital Erlangen, 91054 Erlangen, Germany

**Keywords:** high-dose-rate, interstitial brachytherapy, combined with EBRT, locally advanced superior sulcus tumor, unresectable pancoast tumor, dose escalation

## Abstract

**Objectives**: To analyze the results of interstitial (IRT) high-dose-rate (HDR) brachytherapy (BT) in the primary treatment of patients with unresectable superior sulcus tumors (SST) combined with external beam radiotherapy (EBRT). **Methods**: Between 2013 and 2023, seven patients with unresectable SST were treated with combined BT and EBRT with or without concomitant chemotherapy. The patients’ median age was 64 years (range, 49–79 years) and median tumor volume was 146.8 cm^3^ (range, 29.3–242.3 cm^3^). A median BT dose of 8 Gray (Gy) (range, 5–10 Gy) was prescribed and delivered in a single fraction. A median EBRT dose of 54 Gy (range, 30–59 Gy) was prescribed and administered normofractionated (single dose: 1.8 Gy). **Results**: We report the results of seven patients with SST treated with combined BT and EBRT and followed for a median of 38 months. The overall clinical response rate was 83.33% with five out of six patients achieving local control, while one out of six (16.66%) showed local and general progression. No deaths were attributed to the treatment itself; rather, one patient died during the course of therapy as a result of systemic progression. The most common radiation-related adverse events were grade I–II fatigue and mild paresthesia. No severe toxicity (CTCAE ≥ III°) was observed with interstitial high-dose-rate (HDR) BT combined with EBRT. **Conclusions:** For patients with unresectable superior sulcus tumors, interstitial HDR BT in combination with EBRT is a feasible treatment option that offers the potential for local control and long-term survival. The findings of this study should be validated in a larger patient cohort.

## 1. Introduction

The treatment of superior sulcus tumors (SST) has been established by the results of two comparable prospective studies from the Southwest Cancer Research Group/North American study group [1] and the Japan Clinical Oncology Group [2] showing that preoperative chemoradiotherapy followed by surgery (PCRT-S) resulted in 5-year survival rates of 44% and 56%, respectively. However, the optimal therapeutic approach for primary unresectable tumors remains undefined.

A retrospective monocentric study [3] examined consecutive patients with unresectable superior sulcus tumor non-small cell lung cancer (SST-NSCLC). Patients received induction concurrent chemoradiotherapy (CRT) with cisplatin/vinorelbine/fluorouracil combined with 44 Gray (Gy) radiotherapy (5 fractions of 2 Gy each per week), followed by CRT up to 66 Gy. The median OS in this study was only 9.1 months. The OS rates at one and two years were 45% and 16.9%, respectively. A recurrence was observed in 72% of patients.

The National Comprehensive Cancer Network (NCCN) guidelines recommend stereotactic body radiation therapy (SBRT) as the treatment of choice for dose escalation in patients with unresectable, early-stage, peripherally located NSCLC. [4,5] Subsequently, SBRT was also introduced as a possible treatment option for pulmonary lesions in the oligometastatic setting. [6,7] Based on the premise that dose escalation leads to improved local control, our combined approach of high-dose interstitial (IRT) brachytherapy (HDR) combined with external beam radiotherapy (EBRT) not only seeks to escalate the dose to maximize therapeutic efficacy, but also incorporates precise planning and delivery techniques to carefully protect organs at risk (OARs).

In the present monocentric retrospective analysis, we report our experience with computed tomography (CT)-guided HDR BT in the primary treatment of unresectable SST followed by EBRT and evaluate the safety of interstitial HDR BT.

## 2. Materials and Methods

### 2.1. Patients

We retrospectively analyzed the outcomes of all patients in our institutional database who received treatment for pathologically proven, unresectable superior sulcus tumors. SST was defined as a tumor located on the superior aspect of the lung and involving the apical chest wall above the second rib. From 2013 to 2023, a total of 7 patients underwent interstitial HDR BT in combination with EBRT at our institution. Six out of seven patients were deemed unresectable by the thoracic surgeon at the multidisciplinary tumor board. Only one patient (1/7) with clinical stage T2 (cT2) declined surgical intervention (see Table 1, patient No. 1). The study population included two males and five females with a mean age of 63.7 years (range, 49–79 years). The most prevalent histological subtype was that of squamous cell carcinoma (4/7, 57%) and adenocarcinoma (3/7, 43%). Six out of seven (86%) of the SSTs were classified as clinical stage T4 (cT4). Three out of seven patients (42.9%) received BT and EBRT alone, while four out of seven patients (57.1%) received concomitant EBRT and chemotherapy (cisplatin 20 mg/m^2^, days 1–5, in weeks 1 and 5; vinorelbine 12.5 mg/m^2^, weeks 1–3 and 5–6). The median EBRT dose was 54 Gy administered predominantly with a daily dose of 1.8 Gy. Only 1 out of 7 patients (14%) received subsequent CHT upon progression. Detailed patient characteristics are shown in Table 1.

### 2.2. Treatment Technique

All procedures were performed under general anesthesia with prophylactic antibiotics, in accordance with established techniques [8,9,10], using CT (Siemens Somatom Sensation, Siemens AG, Medical Solutions, Forchheim, Germany) image guidance for interstitial HDR brachytherapy.

Catheters with metal trocars were inserted through the chest wall, guided by CT, to facilitate the treatment of tumors arising directly from the chest wall. This approach ensures safe catheter introduction while taking into account the three-dimensional extent of the tumor. The catheters were strategically placed approximately 1.5–2 cm apart from each other, to optimize the treatment field by ensuring complete coverage of the tumor volume. The median number of catheters was six (range, 2–12). Following catheter placement, spiral computed tomography (CT) was then performed to confirm the treatment planning setup in Oncentra Brachy (versions 4.1 to 4.6), using a 2 mm slice thickness in all cases.

Adjacent critical organs at risk (OARs) and the clinical target volume (CTV) were delineated. The CTV encompassed the gross tumor volume, including the macroscopically visible tumor and presumed tumor extension. No safety margins are incorporated into the calculations. Subsequently, the needle positions were reconstructed to ensure accurate treatment delivery. The system ensured a minimum step size of 0.5 cm for Iridium-192 dosing, with the objective of 98% CTV coverage. Radiation planning was performed according to a standardized data formalism developed by the Interstitial Collaborative Working Group (ICWG) and published by Task Group No. 43 of the AAPM in 1995 (TG-43) [11] and revised in 2004 (TG-43 U1) [12].

Treatment planning aimed to minimize OAR doses while delivering specific dose prescriptions to target volumes. BT was delivered in a single fraction, with a median prescribed dose of 8 Gy (range, 5–10 Gy). The dose to 100% of the CTV ranged from 58% to 89%, with a mean of 75%. The mean dose to 2 cm^3^ of the CTV was 11.1 times higher than the prescribed dose (range, 7.8–13.5).

Following plan approval, it was uploaded to the HDR-afterloading system (microSelectron-HDR, Elekta, Sweden), equipped with an Iridium-192 source, and the patient was irradiated according to the prescribed dose. After treatment, catheters were carefully removed to complete the procedure.

**Figure 1:**
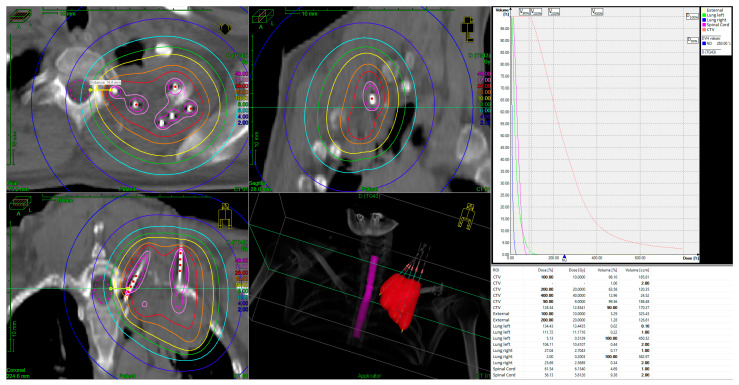
IRT BT implant and dose-volume-histogram (DVH) for an unresectable superior sulcus tumor. The tumor volume was 186 cm^3^, the prescribed HDR dose was 10 Gy, delivered in a single fraction. Color code of the isodose lines: yellow = 100% isodose; orange = 150% isodose; red = 200% isodose; rose = 320% isodose; pink = 400% isodose.

Figure 1 depicts a distribution of a brachytherapeutic dose of a single interstitial boost of 10 Gy administered to patient 3. The localized doses in the vicinity of the needles exceed 40 Gy, while the 15% isodose line (15 Gy) effectively encompasses the clinical target volume (CTV).

External beam radiotherapy (EBRT) commenced between 5 and 18 days after interstitial brachytherapy. EBRT doses ranged from 30 Gy (3 Gy per fraction/10 fractions) to 59.4 Gy (1.8 Gy per fraction/33 fractions), with planning target volumes (PTVs) varying between 571 cm³ and 1052 cm³, with a mean of 792 cm³. The CTV encompassed the GTV, including the primary tumor and the hilar region in cases of lymph node involvement. This delineation was determined by CT and confirmed with PET-CT. An isotropic margin of 1 cm was utilized to expand the CTV to the planning target volume (PTV). Treatment delivery utilized three-dimensional conformal radiotherapy (3D-CRT) or intensity-modulated radiation therapy (IMRT) with image-guided radiation therapy (IGRT), in accordance with international standards [13,14].

### 2.3. Response and Toxicity Evaluation Criteria

Before treatment, all patients underwent comprehensive clinical and neurological evaluations, including assessment using the Karnofsky Performance Scale (KPS). Follow-up consisted of clinical examinations and radiological assessments, adhering to a standardized protocol with initial follow-up at 1.5 months post-therapy, quarterly in the first year, and biannually thereafter. Positron emission tomography (PET) or PET-CT scans were performed annually for radiological monitoring.

Metabolic response was evaluated according to PERCIST criteria [15]. Adverse events were systematically graded using the Common Terminology Criteria for Adverse Events (CTCAE) version 5.0 [16], including fatigue, dysesthesia, dysphagia, pneumonitis, pulmonary fibrosis, mediastinal or pleural hemorrhage, and pneumothorax.

## 3. Results

Our study included seven patients with unresectable superior sulcus tumors who received interstitial HDR BT combined with EBRT. Individualized treatment plans achieved CTV coverage over 98% in six out of seven patients. To avoid overdosing the spinal cord, one patient received a lower dose.

For PTVs exceeding 1000 cm³, EBRT doses were adjusted to account for the increased volume. We calculated the biologically effective dose (BED) and the equivalent dose in 2 Gy fractions using a radiobiology spreadsheet [17]. An α/β ratio of 10 Gy was assumed for the tumor. Further details are provided in Table 2.

Patient characteristics and outcomes are listed in Table 3. Median Karnofsky Performance Scale (KPS) scores improved or remained stable over 12 months, indicating a positive impact on quality of life and symptom management.

**Figure 2:**
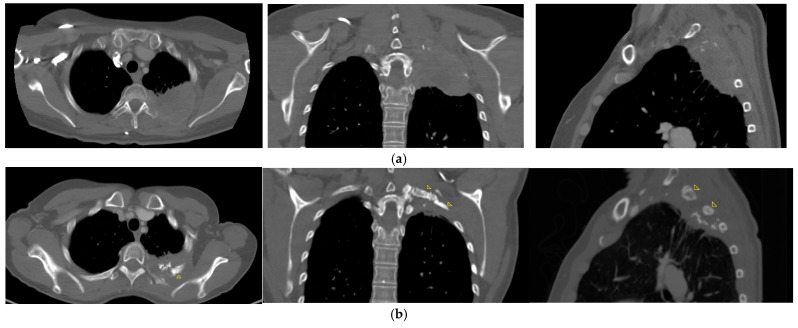
(**a**) Destroyed rib before irradiation. (**b**) Visual evidence of recalcification of rib 36 months after irradiation, indicated by arrows.

As detailed in Table 3, five out of seven patients remain alive and local disease-free for a median follow-up period of 38 months, (range, 31–131 month), demonstrating a complete metabolic response (CMR) on PET-CT scans with excellent quality of life. Of these five patients, three experienced complete pain relief following therapy, having previously reported severe pain. The remaining patients did not experience pain related to their condition. Additionally, two of these patients also regained full arm mobility. Furthermore, patient 3, whose BT plan is also illustrated in Figure 1, exhibited regeneration of a tumor-destroyed rib after 36 months (Figure 2a,b).

Our follow-up findings indicate that no severe adverse events (≥grade III), defined as severe or medically significant, life-threatening, or death related to the adverse event according to the CTCAE, occurred in any of the seven patients treated with interstitial HDR BT. Despite the high locally delivered dose, none of the patients developed a necrotic cavity or other post-therapy complications. A comprehensive list of adverse events is provided in Table 4, the degree symbol (°) represents the grade of severity of adverse events.

Furthermore, Figure 1 elucidates the considerable reduction in dose to adjacent organs at risk, most notably the spinal cord. A reduction in dose from 40 Gy to below 8 Gy was achieved within a distance of 14.9 mm, effectively protecting the spinal cord and enabling the delivery of the required dose for local tumor control. However, as illustrated in the isodoses and the DVH (Figure 1), this technique has been proven to provide effective sparing of the lungs, which is in accordance with the mild to absent occurrence of pneumonitis symptoms.

## 4. Discussion

In locally advanced SST, locoregional control is directly related to improved symptom and pain control, leading to an improved quality of life.

Preoperative chemoradiotherapy followed by surgical resection is the standard of care for localized SST [1,2,18,19,20,21]. However, this approach is not suitable for all patients, particularly those with unattainable technical and functional operability. In such cases, treatment strategies often mirror those for other inoperable lung tumors. [22]. Treatment options for these patients include chemoradiotherapy or radiotherapy alone [23,24]. Improvements in survival rates have been achieved by implementation of targeted therapies in a multimodal approach [25]. Nevertheless, the results are still disappointing.

Recent studies have explored minimally invasive ablative techniques relying on electric or hyperthermic approaches. However, these techniques are associated with high complication rates, and their success is often limited by lesion size. [26,27,28] In contrast to these limitations, interstitial BT offers distinct advantages. Notably, it is not restricted by lesion size, tumor perfusion, or inhomogeneity in tumor tissue. Building upon the established efficacy of interstitial HDR BT in other cancers, this study aimed to evaluate its safety and efficacy in a series of patients with inoperable SST.

Interstitial HDR BT has already proven an effective therapy for the treatment of lung malignancies [29,30,31,32] as well as various tumor entities at other sites like rectal cancer, prostate cancer, soft tissue sarcomas, oral cavity tumors, and glioblastoma [9,33,34,35,36,37,38,39,40]. Interstitial HDR BT constitutes also a possible alternative to EBRT in the adjuvant treatment of breast cancer as partial breast irradiation [41,42], as well as a substantial part of the therapy of locally advanced cervical cancer [43,44,45,46].

Willner et al. were able to find a significant correlation between local tumor control and radiation dose from EBRT doses > 70 Gy in the primary treatment of NSCLC [47]. In locally advanced SST, local control is associated with improved survival. This is supported by a study [48] demonstrating that median survival was significantly prolonged in SST patients who could tolerate high-dose radiation treatment.

In general, long-term control without excessive morbidity is rarely achieved with conventional radiation techniques, as the dose limits for neighboring critical risk organs are too high. A comparison of dose distributions for the irradiation of a tumor in the lung with brachytherapy and a corresponding distribution in teletherapy (3D-conformal technique) reveals that the target volume can be covered similarly well with the 100% isodose in both techniques. However, a key difference lies in the volume of the regions encompassed by the 80% and 60% isodose, which are considerably smaller in brachytherapy than in teletherapy. This helps to protect the lungs and brachial plexus during brachytherapy treatment. In contrast, SBRT has the potential to enhance therapeutic outcomes by increasing treatment doses. The positive results for SBRT indicate a favorable prognosis for local control with proven efficacy in the treatment of small- to medium-sized tumors [49,50]. Depending on OAR in the vicinity, the SBRT-dose is often prescribed at 65–80% of the maximum dose and thus escalating the dose up to 154% of the prescribed dose for a very small volume or point within the PTV. It is possible to prescribe to a lower percentage of maximum, but this leads to a higher dose to adjacent healthy tissue. [51] In interstitial BT, especially in larger volumes, dose escalation beyond 200% within the PTV is easily achieved. Due to the steep dose gradient of the source and to reach the prescribed dose at the PTV surface, relevant volumes surrounding the implanted needles receive significantly higher than the prescribed dose.

A further study has identified the dosimetric benefits of HDR BT over SBRT in the treatment of unresectable hepatocellular carcinoma, particularly in terms of the preservation of uninvolved liver tissue. [52] HDR BT demonstrated a significantly lower mean dose to the liver and a reduction in radiation exposure to non-targeted liver regions, with this advantage being particularly pronounced in cases involving larger tumor volumes.

A study conducted by Bilski et al. offers valuable insights into the dosimetric benefits of HDR BT in comparison to robotic-based and LINAC-based stereotactic body radiation therapy (SBRTck and SBRTe) for liver metastases. [53] It is noteworthy that HDR BT exhibited superior dose coverage within the PTV and delivered the lowest doses to uninvolved liver tissue in comparison to both SBRT techniques. Furthermore, HDR BT demonstrated a reduced dose to critical organs, including the stomach, heart, great vessels, ribs, skin, and spinal cord. This suggests a promising profile for preserving nearby structures. Although SBRTck demonstrated the highest homogeneity index (HI) and SBRTe exhibited the optimal planning conformity index (PCI), HDR BT’s pronounced dose gradients and effective organ at risk sparing. The findings of Bilski et al. highlight the versatility of HDR BT in delivering high radiation doses directly to the target volume with precision, which may offer superior control for larger or irregularly shaped lesions compared to non-invasive SBRT options.

Two other radiotherapy approaches for dose escalation in locally advanced lung tumors are stereotactic body radiotherapy delivered after external beam radiotherapy, often referred to as SBRTpostRT [54], and LATTICE radiotherapy (LRT) [55,56,57], which is a subsequent development of GRID radiotherapy (GRT), delivering a high dose to small subvolumes of PTV. In case of SBRTpostRT, residual disease or gross tumor volume is contoured and boosted. SBRTpostRT offers the advantage of potentially improving local control by delivering a highly conformal boost to the gross tumor volume following initial EBRT tumor size, which should be suitable for SBRT. In our cases, the tumors had been quite bulky and only two out of seven patients had a PTV for BT smaller than 100 cm³; thus, five out of seven were not suitable for SBRTpostRT. For LRT, a distribution of peak dose vertices (spheres) is defined inside the PTV mostly regular separated by 3 cm and 0.5 cm distant from PTV boundary. Placement of vertices is not dependent on any internal structures of PTV. Planning aims to reach a dose five times higher to peaks than to valleys [56,57]. Taking a step back to GRT, these peak dose volumes take the form of long narrow cylinders generated in EBRT. This makes GRT spatially comparable to HDR BT. Around implanted needles, we found in our planes a high dose accumulation of a seven to ten times higher dose than prescribed to GTV, which exceeds the planning aims for LTR [56,57] and GTR [58]. Studies comparing brachytherapy and LRT/GRT would be interesting in the future.

Peters et al. [30] treated 30 patients with 83 primary or secondary lung tumors by single-fraction CT-guided HDR BT. A dose of 20 Gy was prescribed to a mean tumor diameter of 2.5 cm, and the LC rate was 91% at 12 months and 86% at 20 months. Six patients (12% of interventions) developed marginal pneumothoraxes and one patient (1% of interventions) a major pneumothorax. Tselis et al. [59] treated 55 patients with a median tumor volume of 160 cm^3^ with a complete and overall response rate to HDR BT of 18% and 67%, respectively. The overall local tumor control was 88% at 1 year, 81% at 2 years, and 75% at 3 years. Our study builds upon these findings by evaluating the safety and efficacy of interstitial HDR BT in a specific cohort of patients with inoperable SST.

Chatzikonstantinou et al. [8] reported the results of 16 patients with locally advanced, unresectable NSCLC, treated with definitive radiotherapy. Interstitial HDR BT was given as dose escalation to patients with poor response after chemoradiotherapy of 45–50 Gy. Fractionated EBRT has been applied to all patients. The median OS and LC rates after a median follow-up of 12.5 months were 12.9 and 24.9 months, respectively, with an LC rate of 68.9% at one year.

Within our cohort of seven patients, characterized by a median tumor volume of 146.8 cm³ and a median follow-up duration of 38 months, we observed only one tumor-related death due to systemic disease progression during EBRT. The local tumor response among the surviving six patients revealed a promising outcome, with five patients (83.33%) achieving local control of their disease. However, one patient (16.66%) experienced both local and systemic disease progression. These data are comparable to those of other interstitial HDR BT series [60].

Li et al. [61] investigated the risk factors for surgical complications of high-dose 3-dimensional interstitial brachytherapy in lung cancer in a recent study. The most frequent complications of the CT guided HDR BT were pneumothorax and hemorrhage. In particular, the distance between the pleura and the needle, which passes through normal lung tissue, as well as the tumor size and finally the number of needle insertion adjustments were the risk factors. These risk factors are rare in our series of interstitial BT of SST, as the tumor is usually directly associated with the pleura, which lowers the risk of side effects.

In our cohort the overall treatment was well tolerated, without any noteworthy acute or late adverse events observed. Notably, there were no instances of dysphagia, treatment-requiring pneumonitis or pulmonary fibrosis, bleeding, or pneumothorax. Mild fatigue (Grade I) was reported in two out of seven patients, and fatigue grade II in another two out of seven patients, both of which were manageable with rest and did not result in a notable impairment of daily activities. Local dysesthesia was experienced by four out of seven patients, manifesting as mild sensory alteration, while one patient exhibited moderate sensory alteration. Additionally, three out of seven patients demonstrated radiologically confirmed local pneumonitis, which bore no clinical significance. The absence of critical complications or the need for clinical intervention underscores the safety and tolerability of the procedure for patients.

Interstitial HDR BT allows for an unparalleled level of dose customization due to its interstitial placement, directly within the tumor. This method facilitates the delivery of high doses to the tumor while sparing surrounding healthy tissue, making it particularly effective for tumors in anatomically complex locations or those surrounded by sensitive structures. The precision of HDR BT in targeting the tumor allows for an increased dose, potentially leading to better local control and symptom management, such as pain relief and improved mobility, without a proportionate increase in toxicity.

Comparing HDR BT with SBRT is essential, as both offer high precision and dose conformity. While SBRT is less invasive and well-suited for small- to medium-sized tumors in critical locations, it may be limited for very large or irregularly shaped tumors near sensitive structures. This study supports HDR BT as a viable alternative to SBRT, particularly where sparing critical structures is crucial. Further research should explore whether HDR BT’s dosimetric advantages lead to improved clinical outcomes and evaluate its long-term implications across different anatomical locations and clinical settings.

It is important to acknowledge that our BED and EQD2 calculations were performed using standard formulas that assume a homogeneous dose distribution and were in our cases calculated for the prescribed dose to PTV surface. As anticipated in the context of interstitial HDR BT, the actual dose distribution within the target volume was heterogeneous, exhibiting notable dose gradients and substantial dose volumes. This heterogeneity, while allowing for high tumor dose coverage and sparing of surrounding tissues, introduces a degree of uncertainty in the calculated BED and EQD2 values. While these values provide a useful estimate of the biological effect, further research incorporating more sophisticated dosimetric parameters that account for dose heterogeneity is warranted to refine our understanding of the true biological impact of interstitial HDR BT in this setting.

Despite the limitations of our retrospective study design, small cohort size, and variability in treatment regimens, our findings highlight the low toxicity profile of interstitial HDR BT. These promising results advocate for considering interstitial HDR BT as a viable option for non-surgical candidates, particularly in centers skilled in this technique. Further prospective studies with larger cohorts are warranted to confirm these findings and optimize treatment protocols for interstitial HDR BT in SST.

Interstitial HDR BT offers advantages in cases where direct tumor access is possible. This direct access allows for higher dose intensity and improved tumor control while minimizing the risk to adjacent tissues. This is particularly relevant for tumors located near critical structures such as the spinal cord, brachial plexus, or major vessels, where minimizing dose to surrounding tissues is paramount.

## 5. Conclusions

In conclusion, our data indicate that interstitial HDR BT in combination with EBRT appears to be a safe and effective option for dose escalation in locally advanced SST, offering a high degree of intratumoral dose escalation and a steep dose gradient outside the target volume with low toxicity. This approach holds promise for patients who are not suitable for surgery, providing a potentially curative treatment alternative. Nevertheless, our encouraging results warrant a prospective evaluation by a larger patient cohort in prospective trials to define the role of interstitial HDR BT conclusively. The limited sample size of seven patients precludes us from drawing robust statistical conclusions. Instead, our study provides an initial indication of clinical efficacy and safety.

## Figures and Tables

**Table 1:** Patient characteristics.

Patient No.	Age (yrs), Sex	Histological Subtype	TNM	BT: Dose (Gy)	EBRT: Total Dose (Gy)	Tumor Volume at BT (cm^3^)	CHT Simultaneous to EBRT	CHT Upon Progression
1	79, F	SCC	T2 N0 M0	10	54	29.3	+	-
2	74, M	SCC	T4 N2 M1	8	26 *	146.8	-	-
3	50, F	SCC	T4 N0 M0	10	54	185.7	+	-
4	64, M	SCC	T4 N0 M0	5	59	130.4	-	-
5	49, F	ADC	T4 N3 M1	8	59	84.6	+	-
6	67, F	ADC	T4 N0 M0	8	59	149.7	+	-
7	63, F	ADC	T4 N2 M1	5	30	242.3	-	+
mean	63.7			7.7	48.7	138.4		
median	64			8	54	146.8		

* Restriction of therapy due to progressive systemic disease; yrs = years; F = female; M = male; TNM = Tumor—Node—Metastasis classification.

**Table 2:** Tabular list of the biologically effective dose (BED) and the equivalent (isoactive) dose in 2 Gy fractions (EQD2) for BT and EBRT.

Patient No.	BT: Dose (Gy)	EBRT: Total Dose (Gy)	EBRT: Dose per Fraction (Gy)	BED	EQD2	Total BED	Total EQD2
1	10	54	2	64.8	54	84.8	70.7
2	8	26	2	31.2	26	45.6	38
3	10	54	1.8	63.7	53,1	83.7	69.8
4	5	59	1.8	70.1	58,4	77.6	64.7
5	8	59	1.8	70.1	58,4	84.5	70.4
6	8	59	1.8	70.1	58,4	84.5	70.4
7	5	30	3	39	32,5	46.5	38.8
mean	7.7	48.7	2	58.4	48.7	72.5	60.4
median	8	54	1.8	64.8	54	83.7	69.8

**Table 3:** Patient characteristics and outcomes.

Patient No.	KPS Score * PreRT	KPS Score 1.5-Month	KPS Score 12-Month	OS **	LC ***	Patient Alive	Cause of Death	Comments
1	80	70	90	131	131	yes		Painless after IRT BT. No evidence of local recurrence: complete metabolic response (CMR)
2	60	0	0	0	0	no	Rapid progression of systemic disease	Died during EBRT at 26 Gy. (initial distant metastasis)
3	70	60	90	95	95	yes		Clinically significant improvement in pain symptoms and motor skills upon completion of IRT BT. Recalcification of the rib. No evidence of local recurrence (CMR), new singular pulmonary lesion at 54 months after initial therapy, treated with SBRT
4	60	60	0	8	7	no	Progressive systemic disease	Died of local and systemic progression of the disease at 8 months after radiotherapy
5	80	80	80	39	39	yes		Progressive systemic disease (initial distant metastasis) with new cerebral metastasis, under treatment. Locally negative PET-CT scan (CMR)
6	70	60	90	38	38	yes		Clinically significant improvement in pain symptoms upon completion of IRT BT. No evidence of local recurrence (CMR)
7	60	60	60	31	31	yes		Progressive systemic disease (initial distant metastasis), under treatment. Locally negative PET-CT scan (CMR)
mean (*n*)	68.6 (7)	65 (6)	82 (5)	48.9	48.7			
median (*n*)	70 (7)	60 (6)	90 (5)	38	38			

* KPS Score PreRT = on the morning of BT, KPS Score at the 1.5-month and at the 12-month follow-up; ** OS = overall survival in month, from the first diagnosis; *** LC = local control from the date of BT in months; *n* = number.

**Table 4:** Adverse Events post-treatment, categorized according to CTCAE Version 5.0 [16].

Patient No.	Fatigue	Dysesthesia	Dysphagia	Pneumonitis	Pulmonary Fibrosis	Hemorrhage	Pneumothorax
1	-	-	-	-	-	-	-
2	II°	II°	-	-	-	-	-
3	-	I°	-	I°	-	-	-
4	II°	I°	-	-	-	-	-
5	I°	-	-	-	-	-	-
6	-	I°	-	I°	-	-	-
7	I°	I°	-	I°	-	-	-

## Data Availability

The original contributions presented in the study are included in the article, further inquiries can be directed to the corresponding author.
